# Increased copy number at the *HvFT1* locus is associated with accelerated flowering time in barley

**DOI:** 10.1007/s00438-013-0746-8

**Published:** 2013-04-17

**Authors:** Rebecca Nitcher, Assaf Distelfeld, ChorTee Tan, Liuling Yan, Jorge Dubcovsky

**Affiliations:** 1 Department of Plant Sciences, University of California, Davis, Davis, CA 95616 USA; 2 Department of Molecular Biology and Ecology of Plants, Tel-Aviv University, 69978 Tel-Aviv, Israel; 3 Department of Plant and Soil Sciences, Oklahoma State University, Stillwater, OK 74078 USA; 4 Howard Hughes Medical Institute, Chevy Chase, MD 20815 USA

**Keywords:** Vernalization, Barley, *Hordeumvulgare*, Flowering, FT

## Abstract

A precise regulation of flowering time is critical for plant reproductive success, and therefore, a better understanding of the natural variation in genes regulating the initiation of the reproductive phase is required to develop well-adapted varieties. In both monocot and dicot species, the *FLOWERING LOCUS T* (*FT*) is a central integrator of seasonal signals perceived by the leaves. The encoded mobile protein (florigen) is transmitted to the apical meristem where it induces flowering. The *FT* homolog in barley (*Hordeum vulgare* L.), designated *HvFT1,* was shown to correspond to the vernalization locus *VRN*-*H3,* and natural alleles for spring and winter growth habit were identified. In this study, we demonstrate that the *HvFT1* allele present in the barley genetic stock (BGS213) associated with a dominant spring growth habit carries at least four identical copies of *HvFT1*, whereas most barley varieties have a single copy. Increased copy number is associated with earlier transcriptional up-regulation of *HvFT1* and a spring growth habit. This allele is epistatic to winter alleles for *VRN*-*H1* and *VRN*-*H2*. Among accessions with one *HvFT1* copy, haplotype differences in the *HvFT1* promoter and first intron are also associated with differences in flowering time, which are modulated by genetic background. These different *HvFT1* alleles can be used to develop barley varieties adapted to different or changing environments. Our results, together with studies of other wheat and barley flowering genes, show that copy number variation plays an important role in the regulation of developmental processes in the temperate cereals.

## Introduction

Plant reproductive success is highly dependent on a precise targeting of flowering time to a narrow seasonal window that maximizes resources for the developing seeds. In cereal crops, the correct targeting of this optimal reproductive period is translated into increased grain yields. A clear understanding of the natural variation in the genes that regulate flowering time is important to develop varieties adapted to different or changing environments.

In the temperate cereals, which include barley (*Hordeum vulgare* L.) and wheat (*Triticum aestivum* L.), the initiation of the reproductive phase is regulated by the integration of two main seasonal signals: photoperiod (day-length) and vernalization (extended exposures to low temperatures). Based on the response to photoperiod, barley varieties are divided into photoperiod sensitive (accelerated flowering under long days) and insensitive (limited response of flowering time to long days) classes. Based on the response to vernalization, barley varieties are divided into winter (vernalization accelerates flowering) and spring (early flowering irrespective of vernalization) classes.

Most of barley’s natural variation in photoperiod response is associated with allelic differences in the photoperiod genes *PPD*-*H1* and *PPD*-*H2. PPD*-*H1* encodes a pseudo-response regulator (PRR) protein that is part of the circadian clock (Turner et al. 2005) and promotes flowering under long days. Recessive mutations in the *PPD*-*H1* gene reduce expression of *HvFT1* and result in delayed flowering under long days (Hemming et al. 2008; Turner et al. 2005). The *PPD*-*H1* gene acts in conjunction with *CO*-*H1* (Campoli et al. 2012), which is one of the barley homologs of the *Arabidopsis* photoperiod gene *CONSTANS* (*CO*). In *Arabidopsis*, long days result in the stabilization of CO proteins, which up-regulate *FT* resulting in the acceleration of flowering (Corbesier and Coupland 2005). This function seems to be conserved in barley since overexpression of *CO*-*H1* results in the up-regulation of *HvFT1* and the acceleration of flowering (Campoli et al. 2012). The second photoperiod gene, *PPD*-*H2* (*HvFT3*) is a paralog of *HvFT1,* but its effect on flowering is not as strong as *HvFT1* (Kikuchi et al. 2009). The induction of flowering by *PPD*-*H2* seems to be restricted to winter genotypes under non-inductive conditions (short days or long days without vernalization, Casao et al. 2011).

Natural variation in barley vernalization requirement is predominantly found in the vernalization loci *VRN*-*H1*, *VRN*-*H2*, and *VRN*-*H3* (Dubcovsky et al. 2005; Fu et al. 2005; Takahashi and Yasuda 1971; Yan et al. 2005, 2006). The *VRN*-*H1* gene is closely related to the *Arabidopsis* gene *APETALA1,* which encodes a MADS-box protein responsible for the transition of the shoot apical meristem (SAM) from the vegetative to the reproductive stage (Danyluk et al. 2003; Trevaskis et al. 2003; Yan et al. 2003). Deletions of regulatory regions in the *VRN*-*H1* first intron result in a dominant spring growth habit (Fu et al. 2005; Hemming et al. 2009; von Zitzewitz et al. 2005). The second vernalization locus, *VRN*-*H2*, includes three closely related genes characterized by a putative zinc finger and a CCT-domain, designated as *ZCCT* genes (Yan et al. 2004). These genes function as flowering repressors and deletions of all three copies result in spring growth habit independently of the *VRN*-*H1* alleles (Dubcovsky et al. 2005; Yan et al. 2004). Finally, *VRN*-*H3* is a functional homolog of *Arabidopsis* flowering promoting gene *FLOWERING LOCUS T* (*FT*) (Yan et al. 2006) and will be referred hereafter as *FT1* (*HvFT1* in barley and *TaFT1* in wheat).

In both monocot and dicot species, the signals from the vernalization and photoperiod pathways converge at the regulation of *FT1*, which is considered to be a central flowering integrator (Turck et al. 2008). When temperate cereals germinate during the fall, *FT1* is repressed by VRN2, which competes with the photoperiod protein CO for the regulation of *FT1* (Hemming et al. 2008; Trevaskis et al. 2007; Yan et al. 2006, Li et al. 2011). During the winter, vernalization up-regulates *VRN1* (Oliver et al. 2009), which results in the repression of *VRN2* in the leaves and the release of *FT1* transcription in the spring (Loukoianov et al. 2005; Trevaskis et al. 2006; Hemming et al. 2008; Sasani et al. 2009; Chen and Dubcovsky 2012).

In *Arabidopsis* and rice, it has been demonstrated that *FT* encodes a mobile protein (florigen) that travels through the phloem and transmits signals perceived in the leaves to the SAM (Corbesier et al. 2007; Tamaki et al. 2007; Turck et al. 2008). Once FT arrives to the SAM it forms a complex with the bZIP transcription factor FD that physically interacts with the promoters of the meristem identity genes *AP1 (Arabidopsis;* Wigge et al. 2005 *), VRN1* (wheat; Li and Dubcovsky 2008) or *FUL2* (rice; Tsuji et al. 2011), and up-regulates transcription to levels that induce the transition of the SAM to the reproductive stage. In rice, it has been shown that the FT-FD complex also includes 14-3-3 proteins (Taoka et al. 2011).

In barley and wheat, there are other *FT*-like genes (Faure et al. 2007; Kikuchi et al. 2009). However, *FT1* shows the most robust induction of flowering when transformed into rice and wheat (Kikuchi et al. 2009; Yan et al. 2006) and is highly expressed under long days, which indicates that it is likely the key gene in the induction of flowering under long days.

In *Arabidopsis*, the *FT* promoter and first intron have been shown to contain *cis*-regulatory sites that are important for the transcriptional regulation of this gene (Adrian et al. 2010; Helliwell et al. 2006; Schwartz et al. 2009; Tiwari et al. 2010). However, the *FT1* regulatory regions of barley and wheat are not as well characterized. Based on the few sequences available at the time of the cloning of *HvFT1*, Yan et al. (2006) found an association between growth habit and intron haplotypes. However, the sequencing of *HvFT1* alleles from populations previously used to map QTL for flowering time, such as Dicktoo × Morex (Pan et al. 1994) and Sloop x Halcyon (Read et al. 2003; Hemming et al. 2008) failed to reveal any significant association between the intron one haplotypes and flowering time. Inconsistent results were also observed in recent surveys of *HvFT1* allelic variation (Cuesta-Marcos et al. 2010; Casas et al. 2011). In this study, we demonstrate that these inconsistencies were generated by previously unknown copy number variation at the *HvFT1* locus. We found that high *HvFT1* copy number is associated with early flowering, and is epistatic to the *vrn*-*H1* and *Vrn*-*H2* alleles for winter growth habit (independently of *PPD*-*H1*). Finally, once we separated the effects of copy number variation, we were able to better characterize the effect of haplotype variation in *HvFT1* regulatory regions on barley flowering time.

## Materials and methods

### Plant materials

Parental lines used in the different crosses were selected based on their *HvFT1* alleles, including different combinations of promoter and first intron haplotypes. Their growth habits, vernalization and photoperiod alleles, and *HvFT1* haplotypes are indicated in Table 1. All accessions except *H. vulgare* ssp*. spontaneum* Koch (OSU6, PBI004-7-0-015) belong to the sub-species *H. vulgare* ssp. *vulgare*. Hayakiso 2 and Iwate Mensury C (IMC) are commercial barley varieties from Japan and Igri is a variety from Germany, whereas E878 (Ethiopia) and U672 (Russia) are accessions received from the Okayama University barley collection (http://www.shigen.nig.ac.jp/barley/).

**Table 1:** Parental lines used in the different segregating populations

Line	Growth habit	*PPD*-*H1* ^a^	*VRN*-*H1* ^b^	*VRN*-*H2* ^*c*^	*HvFT1* haploid copy number	*HvFT1* haplo. abbr.	Promoter									Intron 1	
							5	4	4	3	2	1	1	1	1	2	3
							4	9	4	9	5	6	5	4	3	7	8
							8	3	1	2	3	7	0	2	7	0	4
BGS213	S	*ppd1*	*vrn1*	*Vrn2*	4	P_E_ I_AG_	4GC	G	A	C	T	i	T	C	C	A	G
IMC	S	*Ppd1*	*vrn1*	*vrn2*	1	P_E_ I_AG_	4GC	G	A	C	T	i	T	C	C	A	G
Morex	S	*ppd1*	*Vrn1*	*vrn2*	1	P_E_ I_AG_	4GC	G	A	C	T	i	T	C	C	A	G
E878	S	*ppd1*	*Vrn1*	*Vrn2*	1	P_E_ I_TC_	4GC	G	A	C	T	i	T	C	C	T	C
U672	S	*ppd1*	*Vrn1*	*Vrn2*	1	P_E_ I_TC_	4GC	G	A	C	T	i	T	C	C	T	C
Hayakiso2	W	*Ppd1*	*vrn1*	*Vrn2*	1	P_E_ I_TC_	4GC	G	A	C	T	i	T	C	C	T	C
Igri	W	*Ppd1*	*vrn1*	*Vrn2*	1	P_L_ I_TC_	8GC	A	G	G	C	d	C	G	T	T	C
*H. v. spont.*	W	*Ppd1*	*vrn1*	*Vrn2*	1	P_L_ I_TC_	8GC	A	G	G	C	d	C	G	T	T	C

Growth habit, alleles at different vernalization genes, and *HvFT1* copy number and haplotypes at the promoter (P_E_ and P_L_) and intron one regions (I_AG_ and I_TC_) are described for each line. Positions of the polymorphisms are reported as bp upstream of the start codon (promoter) or downstream of the start of intron one (intron) in Igri (i = insertion, d = deletion)

^a^ *PPD*-*H1*: the recessive *ppd1* allele is associated with photoperiod insensitivity and the dominant *Ppd1* allele with photoperiod sensitivity

^b^ *VRN*-*H1*: the recessive *vrn1* allele is associated with vernalization requirement (winter growth habit) and the dominant *Vrn1* allele is associated with the lack of vernalization requirement (spring growth habit)

^c^ *VRN*-*H2*: the dominant *Vrn2* allele is associated to vernalization requirement (winter growth habit) and the recessive *vrn2* allele is associated with the lack of vernalization requirement (spring growth habit)

BGS213 is the designated genetic stock for the barley *VRN*-*H3* (=*Sgh3*) allele associated with a dominant spring growth habit (Franckowiak and Konishi 1997). BGS213 is a line derived from the cross Tammi × Hayakiso 2 (GSHO 764) which has been selected for the dominant *Vrn*-*H3* allele from Tammi (early flowering variety from Finland) and for recessive *vrn*-*H1* and dominant *Vrn*-*H2* alleles for winter growth habit (Takahashi and Yasuda 1971). Two additional sets of spring *VRN*-*H3* isogenic lines were developed by backcrossing the *VRN*-*H3* allele from Tammi into the winter varieties Hayakiso 2 and Dairokkaku 1 for eleven generations (Takahashi 1983), designated hereafter as Hayakiso 2-Tammi and Dairokkaku 1-Tammi.

### Haplotypes

Based on a limited number of barley lines, it was initially hypothesized that haplotype variation in *HvFT1* was associated to differences in flowering time (Yan et al. 2006). To facilitate the description of the different haplotypes used in this study, we assigned names to each of the haplotypes found in the promoter and intron one regions. The promoter haplotype present in E878, U672, BGS213, Hayakiso 2, and IMC is referred hereafter to as P_E_ (promoter-early) whereas the promoter haplotype in Igri and *H. vulgare* ssp*. spontaneum* is referred to as P_L_ (promoter-late). These two promoter haplotypes differ in nine linked SNPs and indels (Table 1). The two linked SNPs in the first intron are used to name the intron haplotypes as either I_TC_ or I_AG_. Copy number variation is indicated separately in Table 1.

### Segregating populations and growing conditions

A summary of the crosses and segregating populations included in this study is available in Table 2. The two crosses between BGS213 and winter lines *H. vulgare* ssp*. spontaneum* and Igri were published before (Yan et al. 2006) but are re-analyzed here in the light of the new copy number variation in *HvFT1* presented in this study. Two additional crosses include the varieties Morex (Morex × *H. vulgare* ssp*. spontaneum*) and IMC (IMC × Hayakiso 2), which have identical *HvFT1* haplotypes as BGS213 but different copy number. A third cross was made between BGS213 and IMC to test the effect of *HvFT1* copy number variation on flowering time in lines with identical promoter and intron one haplotypes.

**Table 2:** Segregating populations

Parental lines	CNV	*HvFT1* alleles	Number of plants tested (selected alleles)
BGS213 × *H. vulgare* ssp. *spontaneum* ^a^	4 × 1	P_E_ I_AG_ × P_L_ I_TC_	72 (F_2_)
BGS213 × Igri^a^	4 × 1	P_E_ I_AG_ × P_L_ I_TC_	96 (F_2:3_)
BGS213 × IMC	4 × 1	P_E_ I_AG_ × P_E_ I_AG_	164 (F_2_, *Vrn*-*H2/*−*, Ppd*-*H1/*−)
Morex × *H. vulgare* ssp. *spontaneum*	1 × 1	P_E_ I_AG_ × P_L_ I_TC_	81 (F_2_)
IMC × Hayakiso 2	1 × 1	P_E_ I_AG_ × P_E_ I_TC_	70 (F_2_, *Vrn*-*H2/*−)
E878 × *H. vulgare* ssp. *spontaneum*	1 × 1	P_E_ I_TC_ × P_L_ I_TC_	47 (F_3_, *Ppd*-*H1*) 89 (F_3_, *ppd*-*H1*)
U672 × *H. vulgare* ssp. *spontaneum*	1 × 1	P_E_ I_TC_ × P_L_ I_TC_	42 (F_2_)
Hayakiso 2 × *H. vulgare* ssp. *spontaneum*	1 × 1	P_E_ I_TC_ × P_L_ I_TC_	125 (F_2_) + 134 (F_2_)

Crosses made to study the effect of different combinations of *HvFT1* haplotypes and copy number variation (CNV) on heading time

^a^Yan et al. 2006

Finally, the effect of the different *HvFT1* promoter haplotypes in varieties with identical *HvFT1* copy number was tested in two F_2_ segregating spring x winter populations from crosses E878 × *H. vulgare* ssp*. spontaneum* and U672 × *H. vulgare* ssp*. spontaneum* and in one winter x winter population from the cross Hayakiso 2 × *H. vulgare* ssp*. spontaneum.* The effect of the different intron one haplotypes was only explored in the IMC × Hayakiso 2 segregating population.

All populations were produced and grown in greenhouse under long day (LD) photoperiod (15–16 h day length) generated by extending natural light conditions with supplementary lights as needed. Temperatures were held at non-vernalizing conditions (21–25 °C during the day and 12–18 °C during the night).

### Markers

The *Vrn*-*H1* and *vrn*-*H1* alleles were identified using the *UCW132* marker (Table 3), which detects a small indel near the end of the first intron that is linked to the larger functional deletion in the same intron. The two recessive *vrn*-*H1* alleles in the Hayakiso 2 × *H. vulgare* ssp*. spontaneum* population were characterized using HvV1PromF2 and HvV1PromR2 primers that detect a promoter indel (Table 3). *PPD*-*H1* alleles were identified using a Cleaved Amplified Polymorphic Sequence (CAPS) marker digested with restriction enzyme *Bst*UI (Table 3; Turner et al. 2005). *VRN*-*H2* was genotyped using primers VRN-H2aF and VRN-H2aR (Table 3), or when a codominant marker was necessary, with a marker for the tightly linked *SNF2* gene (Table 3). The *HvFT1* marker based on a 4-bp indel in the promoter was used for genotyping the populations segregating for the *HvFT1* promoter haplotypes (Table 3; Yan et al. 2006), whereas the *UCW133* marker was used to differentiate the intron haplotypes (Table 3).

**Table 3:** Primer names, sequence, amplification temperatures, and amplification efficiencies

Genotyping					
Gene	Primer name	Forward primer	Reverse primer	A.T.	Enzyme
*Vrn*-*H1*	UCW132_F/R	TGTTTTGCAAACTATTTGACCAG	TAGCGCTCATACCGTTCAAG	59°	–
*vrn*-*H1*	HvV1PromF2/R2	ACTTCACCCAACCACCTGAC	CTGGCGGTTGATCTTGTTCT	55°	–
*Vrn*-*H2*	VRN-H2a_F/R	CATGAAACAGCAGCTCCAGA	TTTGCCTCTCTCTCCTGCAT	59°	–
*SNF2*	SNF_F/R	TTGGTACTTGAATGCCTGAAAA	ATGGCACAACTTGGATTTGA	60°	–
*Ppd*-*H1*	PPD/H1/F/R	CTGAGCCTGAAGAGGTCGAG	GTGGCGGGAGGTTATCTCT	57°	*Bst*UI
*HvFT1* Prom.	HvFT1F/R1	ATGGACATGGAACCTGCCACT	TGGTGATGATGAGTGTTGCCC	55°	–
*HvFT1* Intron	UCW133_F/R	TGCACACACTTAGCGCAGTA	GCAGACCGTGGAACTCAACT	55°	*RsaI*
EBmac603	EBmac603F/R	ACCGAAACTAAATGAACTACTTCG	TGCAAACTGTGCTATTAAGGG	56°	–
Bmag914	Bmag914F/R	GGGCAATATACAGTTCAACTC	ATGAACTGGAGGCAGTAAATA	57°	–

qRT-PCR^a^				
Gene	Primer name	Forward primer	Reverse primer	Efficiency %
*UCW118*	CNV_UCW118_F1/R1	CAGTAAGGCGAACCATGTCATC	TGCGCACCAACACAGAACA	99.9
*FT1*_Prom.	CNV_FT1p_F2/R2	CGGCCGAGTCTGTGTGATCT	GGCATAAATCCCGCCTCTTT	99.9
*FT1*_ATG	CNV_FT1p_F5/R4	TGTTCTAAGAAGGAAGGAGAAATGG	GAAGGTCACCCTGAGGTTGGT	99.8
*FT1*_Ex1	CNV_FT1_F2/R2	CGTACGTACACAATCACCACTATCTAATG	GAGAGCCCGATCGTGCAT	99.8
*FT1*_Ex3	CNV_FT1_F4/R3	GCAGGTTGGTGACAGATATCCC	GGAAGAGCACGAGCACGAA	89.3
*UCW123*	CNV_UCW123_F1/R1	ACTGCAAGAGCTACAGCCTTCA	GTCACCGGCAGCAAGATCTAG	99.9
*UCW120*	CNV_UCW120_F1/R1	GCGACGACCAGTAAAAAATGC	CCGTTTCCGTGGATGGAA	99.9
*SNF2*	CNV_SNF2_F1/R1	ATTACCGCTCTGCTGTCGCGATTA	AAATGTGGCTCTGAAGGTGTTGGC	93.4

^**a**^PCR program:(95° 20 s)  × one cycle, (95° 3 s, 60° 30 s) × 40 cycles

### Determination of copy number variation (CNV) by quantitative PCR

Genomic DNA was extracted using the CTAB (cetyltrimethylammonium bromide) extraction method (Murray and Thompson 1980). Samples were treated with RNAse A, and a phenol:chloroform:isoamyl alcohol purification step was used to remove possible RNA contamination, which might interfere with normalization. DNA concentration was normalized using a Nanodrop instrument (Thermo Fisher Scientific, Waltham, MA) to a concentration of 20 ng/μl, and 1 μl was used for each 20 μl Fast Sybr^®^Green reaction. These reactions were performed on an AB7500 Fast Real-Time PCR System (Applied Biosystems by Life Technologies, Grand Island, New York), using identical programs for all primer pairs ((95° 20 s) × one cycle, (95° 3 s, 60° 30 s) × 40 cycles). The parental lines were tested with various pairs of primers in the *HvFT1* region (Table 3), and a pair of primers in the *SNF2* control gene, which has been shown before to have a single copy in the barley genome (Yan et al. 2002). The 2^−ΔΔCт^ method was used to estimate copy number (Weaver et al. 2010), with Morex as a calibrator and C_T_ = threshold cycle. We first calculated ΔMorex = Morex–*HvFT1* C_T_−Morex–*SNF2* C_T_ and ΔTarget = Target–*HvFT1* C_T_−Target–*SNF2* C_T_, and then calculated the difference between the two as −ΔΔC_T_ = ΔMorex−ΔTarget. Previous sequencing of Bacterial Artificial Chromosomes (BACs) containing *HvFT1* has shown that only one copy of this gene is present in Morex, making it a good calibrator variety (Yan et al. 2006). Table 3 describes the primers used for quantitative PCR and their efficiency.

The first *HvFT1* primer pair (CNV_FT1_F2 and CNV_FT1_R2) was designed on the border between the first exon and the first intron. This region was selected because it includes several SNPs that differentiate *HvFT1* from other members of the *FT* family. Copy number in the recombinant BGS213 × IMC F_2_ lines was determined using these primers.

To determine the borders of the duplication in the BGS213 lines, additional primers were designed inside and flanking the *HvFT1* gene. In the *HvFT1* promoter region, a set of primers was designed 656–727 bp upstream of the start codon (CNV_FT1p_F2 and CNV_FT1p_R2). Another set of primers amplified a region from a 22-bp upstream to 97-bp downstream of the start codon (CNV_FT1p_F5 and CNV_FT1p_R4). Within the gene, an additional set of primers was designed at exon three to determine if the complete gene was duplicated (CNV_FT1_F4 and CNV_FT1_R3, 778-902 bp downstream from the start codon). Markers were also designed outside the *HvFT1* gene. One pair of primers was designed for gene *UCW123*, located 6.6 kb downstream from *HvFT1* on Morex BAC clone 440G4 (GenBank DQ900686, CNV_UCW123_F1 and CNV_UCW123_R1). The nearest known genes on flanking BACs 455J22 (GenBank DQ900687) and 761F04 (GenBank DQ900685, Yan et al. 2006) were also tested, including *UCW120* (CNV_UCW120_F1 and CNV_UCW120_R1) and *UCW118* (CNV_UCW118_F1 and CNV_UCW118_R1).

### *HvFT1* expression profiles

To test the effect of the dosage of the duplicated *HvFT1* locus, the isogenic lines of Hayakiso 2 with and without the Tammi *HvFT1* allele were intercrossed and the F_2_ lines were genotyped for *HvFT1* and then tested for *HvFT1* expression by quantitative RT-PCR (qRT-PCR) with Fast Sybr^®^Green Master Mix on the AB7500 Fast Real-Time PCR System (Yan et al. 2006). *ACTIN* was used as an endogenous expression control (Trevaskis et al. 2006).

## Results

### Variation in flowering time among lines with identical *HvFT1* haplotypes

The *HvFT1* sequences from Morex, IMC, and BGS213 are identical (Table 1). However, segregating populations including the first two varieties (Morex × *H. vulgare* ssp*. spontaneum* and IMC × Hayakiso 2) showed that plants with winter alleles for the *VRN*-*H1* and *VRN*-*H2* genes flowered very late independently of *HvFT1*. This observation was inconsistent with the results from Yan et al. (2006), which have previously shown that the *HvFT1* allele from BGS213 was sufficient to confer a spring growth habit when introgressed into winter barley varieties. To test if additional polymorphism were present among these three *HvFT1* alleles, we sequenced a 9,250-bp region starting 1,499-bp upstream of the start codon and ending 6,690-bp downstream of the stop codon. No polymorphisms were detected in the *HvFT1* coding and flanking sequences of Morex, IMC, and BGS213. The segregating populations including these varieties are described in detail below.

#### Morex (P_E_ I_AG_) × *H. vulgare* ssp*. spontaneum* (P_L_ I_TC_)

This population of 81 F_2_ plants segregated for the *VRN*-*H1, VRN*-*H2*, and *HvFT1* loci. To study the effect of the *HvFT1* alleles in a winter background, we first selected plants homozygous for *vrn*-*H1* and homozygous or heterozygous for the *Vrn*-*H2* alleles for winter growth habit. The 11 selected plants carrying these alleles flowered relatively late (91–106 days after sowing) and showed no significant differences between the different *HvFT1* alleles (*P* = 0.32), even when the 11 plants were divided in photoperiod sensitive and photoperiod insensitive groups.

#### IMC (P_E_ I_AG_) × Hayakiso 2 (P_E_ I_TC_)

A similar result was observed in a population of 92 F_2_ lines from the cross between the photoperiod sensitive varieties IMC and Hayakiso 2. IMC is a spring barley variety that carries an *HvFT1* allele with identical sequence to the BGS213 allele and a *vrn*-*H2* allele for spring growth habit, whereas Hayakiso 2 is a winter variety (Table 1). In the F_2_ segregating population, 22 plants homozygous for the IMC *vrn2* allele were identified using the tightly linked *SNF2* molecular marker and were discarded. The remaining 70 plants, carrying *vrn*-*H1* and *Vrn*-*H2* alleles for winter growth habit, flowered relatively late (100–250 days, average 167 days) and showed no significant differences between the different *HvFT1* alleles (*P* > 0.05). Plants homozygous for the functional *Vrn*-*H2* allele flowered 29 days later than the plants carrying the heterozygous allele (*P* = 0.02) suggesting some dosage effect of *VRN*-*H2.* No significant interaction was detected between *HvFT1* and *VRN*-*H2* (*P* = 0.74).

The results from the IMC × Hayakiso 2 and Morex × *H. vulgare* ssp*. spontaneum* populations suggest that, in spite of their identical sequence, the *HvFT1* alleles present in Morex and IMC have a smaller effect on inducing flowering than the *HvFT1* allele present in BGS213. To test this hypothesis, we developed a third population segregating for the *HvFT1* alleles of BGS213 and IMC, which have identical sequence and contrasting phenotypes.

#### BGS213 (P_E_ I_AG_) × IMC (P_E_ I_AG_)

Since no polymorphisms were found between the two parental lines in *HvFT1* or in the genes tightly linked to *HvFT1* (Yan et al. 2006), we screened linked SSR markers and found *Bmag914* and *EBmac0603* (GrainGenes database, http://wheat.pw.usda.gov/) to be polymorphic between BGS213 and IMC. These two markers flanking the *HvFT1* locus were located approximately 14 cM apart in this mapping population.

The complete segregating population was genotyped with the *HvFT1* flanking SSR markers and with markers for *VRN*-*H2* and *PPD*-*H1* genes. To focus on the effect of *HvFT1*, plants homozygous for the *vrn*-*H2* allele for spring growth habit or for the photoperiod insensitive allele (*ppd*-*H1*) were eliminated. The remaining 167 F_2_ lines showed a highly significant (*P* < 0.0001) effect on flowering associated with the SSR markers flanking the *HvFT1* locus. The *HvFT1* locus alone explained 96 % of the variation in flowering time among the selected 167 plants, indicating that no other major flowering gene was segregating in this selected sub-population.

Plants heterozygous or homozygous for the BGS213 *HvFT1* allele showed a spring growth habit (33 days to 76 days from sowing to heading time) whereas plants homozygous for the IMC SSR markers flanking the *HvFT1* locus showed no signs of flowering 125 days after planting, when the experiment was terminated. This population showed a clear 3:1 ratio between spring and winter plants (122 spring/45 winter, *χ* ^2^ *P* = 0.56). In summary, these results confirmed that the largest differences in flowering time in this segregating population were linked to the *HvFT1* region in spite of the identical *HvFT1* sequences between BGS213 and IMC.

### Variation in *HvFT1* copy number

Possible explanations for the previously described differences in flowering time include an unknown gene tightly linked to *HvFT1*, a regulatory sequence beyond the *HvFT1* sequenced region or copy number variation (CNV) in *HvFT1*. To test the last hypothesis, we determined the number of *HvFT1* copies in BGS213, IMC, Morex, and other accessions used as parental lines in different segregating populations.

**Fig. 1:**
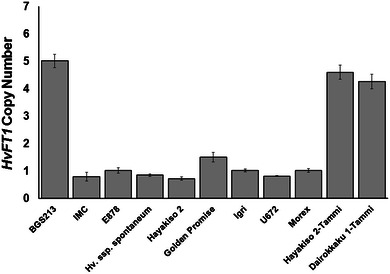
*HvFT1* haploid copy number is increased in BGS213. Copy number was determined using the 2^−ΔΔCT^ method (Weaver et al. 2010) and primers for the first exon (FT1_Ex1, Table 3). The single copy gene *SNF2* was used as internal control and the variety Morex as calibrator (see “Materials and methods”). Averages and standard errors of the means are based on 7–10 biological independent DNA extractions

DNAs were extracted from approximately 10 plants from each variety, and *HvFT1* copy number was determined by quantitative PCR for the exon one region (Table 3) using the single copy gene *SNF2* as an internal reference and Morex as a calibrator (see Materials and Methods). The results indicated that BGS213 has four to five copies of *HvFT1* whereas IMC has a single copy (Fig. 1). The increase of *HvFT1* copy number found in the BGS213 allele was confirmed in the two backcross substitution lines of the Tammi *HvFT1* allele into the winter varieties Hayakiso 2 and Dairokkaku 1, which also showed four to five copies of *HvFT1* each. All other varieties tested in this experiment (E878, *H. vulgare* ssp*. spontaneum*, Hayakiso 2, Golden Promise, Igri, U672 and Morex) showed only one copy of *HvFT1.*

**Fig. 2:**
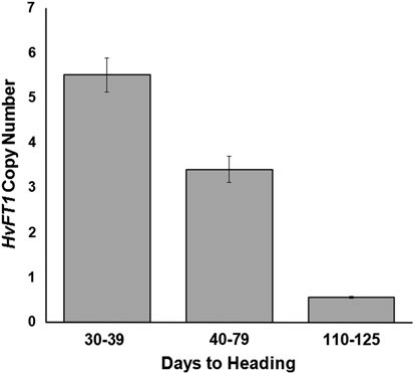
Co-segregation of *HvFT1* copy number and heading time in 45 F_2_ lines from the BGS213 × IMC population selected for recombination between SSR markers flanking the *HvFT1* gene. Late flowering lines showed a single *HvFT1* copy, lines with intermediate flowering showed on average ~3 copies, and early flowering lines showed an average of ~5 copies. Haploid copy number was determined as described in Fig. 1

To test if the multiple *HvFT1* copies were linked, we estimated *HvFT1* copy number with the same exon one primers in 45 BGS213 x IMC F_2_ lines selected for recombination between the *HvFT1* flanking markers *Bmag914* and *EBmac0603.* The lines that flowered earlier (30–39 days) showed the highest average copy number (~5 copies), whereas the lines that flowered later (>110 days) showed an average of one *HvFT1* copy. Plants with intermediate flowering times are likely heterozygous since they showed an intermediate copy number (~3 copies). These results confirmed that *HvFT1* copy number co-segregates with flowering time (Fig. 2), and that the different *HvFT1* copies are linked.

**Fig. 3:**
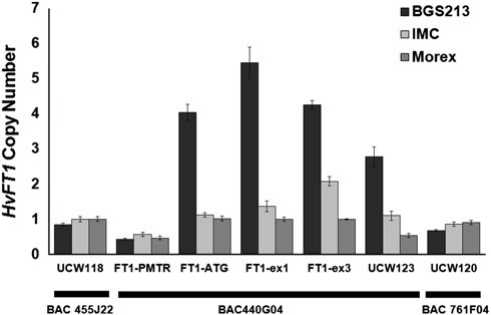
Extension of the *HvFT1* duplicated region in BGS213. Primers in the flanking BACs (Yan et al. 2006) and the *HvFT1* promoter (*FT1*-PMTR, 655–700 bp upstream of start codon) showed no evidence of duplication. Primers flanking the start codon (*FT1*-ATG), in the first exon (*FT1*-ex1), in the third exon (*FT1*-ex3) and in the *UCW123* marker 6.6-kb downstream of the stop codon showed evidence of duplication. Averages and standard errors of the means are based on 7–10 independent DNA extractions

We performed an additional experiment to determine the extension of the duplicated region. Primer pairs within loci *UCW118* and *UCW120* located in Morex’s *HvFT1* flanking BACs 455J22 and 761F04 (Yan et al. 2006), showed no evidence of duplication in BGS213 (Fig. 3). These results indicated that the duplication of the *HvFT1* region did not include the adjacent BACs. Therefore, we designed additional primers within the Morex BAC 440G04, which includes the *HvFT1* gene. The primer set for the *HvFT1* promoter region located 655–700 bp upstream of the ATG start site showed no increase in copy number (Fig. 3). Since the start codon region shows four to five copies, the duplicated region must start within the 600-bp region of the *HvFT1* promoter upstream of the start codon. The primers for the third exon of *HvFT1* and for the *UCW123* marker located 6.6-kb downstream from *HvFT1* showed that the duplicated region extended beyond the coding region of *HvFT1* and into this marker (Fig. 3).

### Differences in *HvFT1* expression in alleles with different copy number

To study the relationship between *HvFT1* copy number variation and expression, we crossed Hayakiso 2 with its near isogenic line containing the Tammi allele (Hayakiso 2-Tammi), selected eight plants homozygous for each of the parental alleles and eight heterozygous plants from the segregating F_2_ population, and characterized *HvFT1* expression profiles in the leaves by qRT-PCR.

**Fig. 4:**
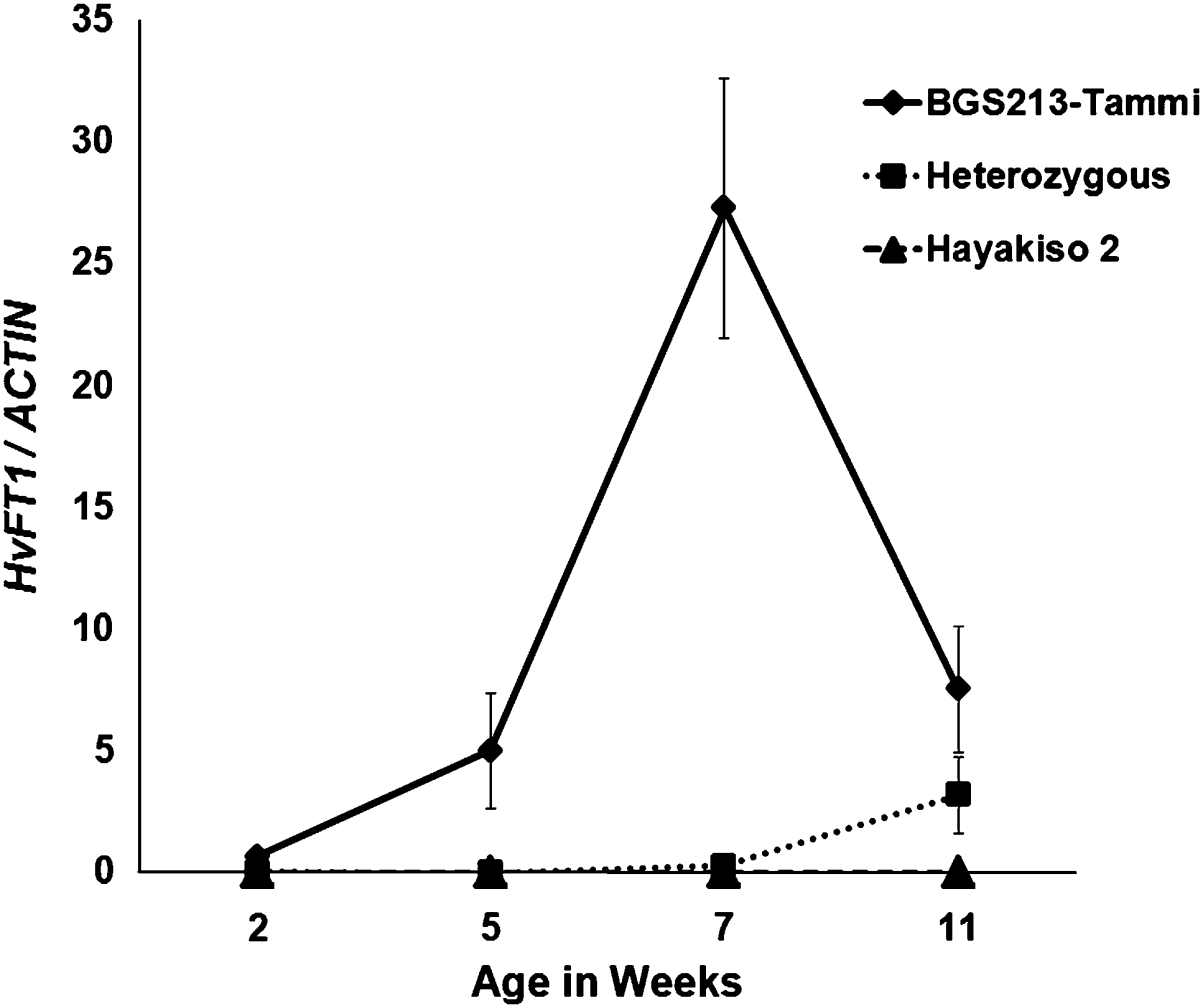
*HvFT1* expression profiles correlate with copy number in a segregating population from the cross between isogenic lines Hayakiso 2 and Hayakiso 2-Tammi. Plants homozygous for the Tammi allele (*black solid line* with *black diamonds*) showed earlier induction of *HvFT1* than those homozygous for Hayakiso 2 allele (*dashed black line* with *triangles*). In the heterozygotes plants (*dotted black line* with *squares*) *HvFT1* transcript levels started to be induced by the end of this time course experiment. *Bars* are plus-minus one standard error of the means

Hayakiso 2-Tammi lines showed earlier expression of *HvFT1*, with significantly higher transcript levels of *HvFT1* expression than the two other genotypes just 2 weeks after planting (three leaf stage; Fig. 4, *P* = 0.002). Plants homozygous for the Tammi *HvFT1* allele flowered around seven to 8 weeks, after which *HvFT1* transcripts fell back to lower levels. The heterozygous plants showed an increase of *HvFT1* transcript levels at the last time point of the experiment (11 weeks), when approximately one-third of the plants had already headed. At this same time point, plants homozygous for the *HvFT1* alleles from Hayakiso 2 showed no detectable levels of *HvFT1* and no signs of flowering induction (Fig. 4). These results show that the transcript levels of *HvFT1* are affected by copy number, with larger copy number resulting in earlier *HvFT1* expression and early flowering.

### Association between differences in *HvFT1* haplotypes and heading time

Since *HvFT1* copy number differences have such a large effect on flowering time, the effect of the different *HvFT1* haplotypes needs to be tested using varieties with the same copy number.

Only one population was available to study the effect of the *HvFT1* first intron haplotypes in lines with a single *HvFT1* copy and identical *HvFT1* promoter haplotypes (Table 1).

#### IMC (P_E_ I_AG_) × Hayakiso 2 (P_E_ I_TC_)

Both IMC and Hayakiso 2 are photoperiod sensitive, and carry a single copy of *HvFT1* with the same promoter haplotype, but they differ in their first intron haplotypes (Table 1). Plants homozygous for the I_AG_ allele flowered on average 10 and 13 days later than the plants heterozygous and homozygous for the I_TC_ allele, respectively. However, these differences were not significant (*P* = 0.19 within the *VRN2* heterozygous class) likely due to the large variability of late flowering plants (100–250 days) that resulted in a reduced statistical power. The late flowering observed in all plants indicates that none of the *HvFT1* alleles segregating in this population was epistatic to the *VRN*-*H1* and *VRN*-*H2* alleles for winter growth habit.

Three additional populations were used to study the effect of the *HvFT1* promoter haplotypes in lines with a single *HvFT1* copy (Fig. 3) and identical *HvFT1* intron one haplotypes (Table 1).

#### E878 (P_E_ I_TC_) × *H. vulgare* ssp. *spontaneum* (P_L_ I_TC_)

This F_2_ population segregated for *HvFT1, VRN*-*H1* and *PPD*-*H1*. To simplify the analysis, two separate F_3_ segregating populations were derived in which the photoperiod sensitive (*Ppd*-*H1*) and photoperiod insensitive (*ppd*-*H1*) alleles were fixed. The ANOVA models including the *VRN1* and *HvFT1* loci, and their interactions explained 92 and 94 % of the variation in heading time in the photoperiod sensitive and insensitive subpopulations, respectively, which indicates that these two genes account for most of the variation in heading time in these two sub-populations. Both sub-populations showed highly significant interactions between the *VRN*-*H1* and *HvFT1* loci (*P* < 0.0001) and therefore, the effects of the *HvFT1* alleles on heading time are described separately for the recessive and dominant (homozygous plus heterozygous) *VRN*-*H1* classes.

**Fig. 5:**
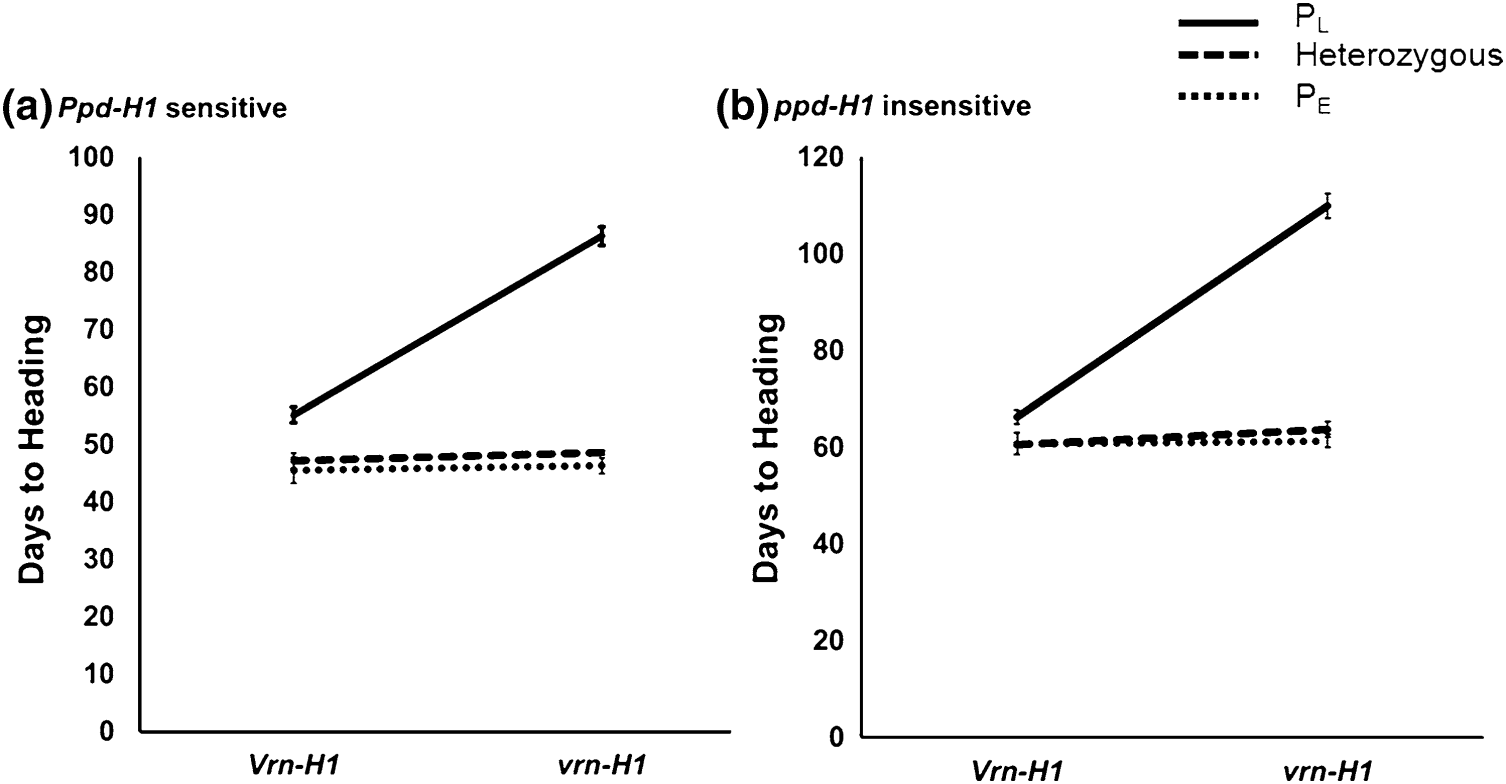
*VRN*-*H1* and *HvFT1* alleles show significant interaction in E878 (P_E_ I_TC_) x *H. vulgare* ssp. *spontaneum* (P_L_ I_TC_) populations segregating for the *HvFT1* promoter haplotypes. **a** Photoperiod sensitive F_3_ family, **b** photoperiod insensitive F_3_ family. The lack of parallelism between *lines* reflects the significant interaction between *VRN*-*H1* and *HvFT1* alleles (larger effect of the *HvFT1* alleles within the *vrn*-*H1* class than within the *Vrn*-*H1* class). *Bars* are plus-minus one standard error of the means

The statistical analysis of the photoperiod sensitive F_3_ family (47 plants, Fig. 5a) showed significant effects of the *HvFT1* alleles within both the dominant *Vrn*-*H1* class (9.5 days, *P* = 0.0031) and the recessive *vrn*-*H1* class (39 days, *P* < 0.0001). Here, the differences in heading time were approximately fourfold larger within the recessive *vrn*-*H1* class than within the dominant *Vrn*-*H1* class. The *HvFT1* allele from E878 was associated in both cases with early flowering.

The plants from the photoperiod insensitive F_3_ family (89 plants, Fig. 5b) flowered on average 20 days later than the photoperiod sensitive F_3_ family (Fig. 5a). As in the photoperiod sensitive plants, the effect of the *HvFT1* promoter haplotypes was larger among the plants homozygous for the recessive *vrn*-*H1* allele (48.5 days, *P* < 0.0001) than among those homozygous or heterozygous for the dominant *Vrn*-*H1* allele (5.5 days, *P* = 0.0002). As in the previous family, the *HvFT1* P_E_ haplotype from E878 was associated with early flowering.

The reciprocal epistatic effect was observed when the effect of the *VRN*-*H1* alleles was studied within each of the *HvFT1* classes. No significant differences in heading time were detected between *VRN*-*H1* alleles among the plants homozygous for the *HvFT1* P_E_ promoter allele, but large differences were observed among the plants homozygous for the *HvFT1* P_L_ promoter allele, both in the photoperiod sensitive (30 days, *P* < 0.0001) and the photoperiod insensitive sub-populations (43.6 days, *P* < 0.0001).

#### U672 (P_E_ I_TC_) × *H. vulgare* ssp. *spontaneum* (P_L_ I_TC_)

Similar results were observed in an additional small F_2_ population (42 plants) segregating for the promoter haplotypes. In this population, the effect of the *HvFT1* alleles was also larger within the plants homozygous for the *vrn*-*H1* allele for winter growth habit (17 days difference, *P* = 0.008) than among the plants including a dominant *Vrn*-*H1* allele in homozygous or heterozygous state (6 days difference, *P* = 0.10). In both classes, the P_E_ allele was associated with early flowering.

In both E878 and U672 populations, the *HvFT1* P_E_ I_TC_ haplotype was associated with early flowering and was epistatic to the winter *VRN*-*H1* and *VRN*-*H2* alleles. However, the same P_E_ I_TC_ haplotype is also present in the variety Hayakiso 2 (Table 1), which has a winter growth habit. To test if this effect was caused by unlinked epistatic genes in Hayakiso 2 or by a specific characteristic of the *HvFT1* region in Hayakiso 2, we crossed this variety with *H. vulgare* ssp*. spontaneum* to generate a third population segregating for the *HvFT1* promoter haplotype. These two varieties are photoperiod sensitive and have a winter growth habit.

#### Hayakiso 2 (P_E_ I_TC_) × *H. vulgare* ssp*. spontaneum* (P_L_ I_TC_)

This population was characterized in two separate experiments performed in California (125 F_2_) and Oklahoma (134 F_2_). The effect of the *HvFT1* alleles was consistent across experiments (no significant experiment × *HvFT1* interaction), so the results from the two experiments were analyzed in a single factorial ANOVA including experiment, *VRN*-*H1* and *HvFT1* as factors. This analysis showed significant differences in heading time between the two recessive *vrn*-*H1* alleles (*P* < 0.0001) and also between the *HvFT1* P_E_ and P_L_ alleles (*P* < 0.0001), but no significant interaction between the two loci (*P* = 0.36). On average, F_2_ plants carrying the *vrn*-*H1* allele from *H. vulgare* ssp*. spontaneum* (average 106 days after sowing) flowered 31 days earlier than those carrying the corresponding allele from Hayakiso 2 (135 days after sowing, Table 4). These results indicated that even though both varieties have recessive *vrn*-*H1* alleles for winter growth habit, the *vrn*-*H1* region from Hayakiso 2 is associated with a stronger vernalization requirement than the one from *H. vulgare* ssp*. spontaneum.* The earlier *vrn*-*H1* allele was dominant, since the difference in heading time between plants heterozygous and homozygous for the *H. vulgare* ssp*. spontaneum VRN1* allele was less than 1 day (Table 4). Surprisingly, plants carrying the *HvFT1* allele from Hayakiso 2 (P_E_ promoter) were on average 10 days later than the heterozygous plants and 14 days later than the plants homozygous for the *HvFT1* allele from *H. vulgare* ssp*. spontaneum* (P_L_ promoter), and the differences were highly significant (*P* < 0.0001, Table 4).

**Table 4:** Hayakiso 2 (P_E_ I_TC_) × *H. vulgare* ssp. *spontaneum* (P_L_ I_TC_): average heading time plus-minus standard error of the means

*VRN*-*H1* allele^a^	*HvFT1* allele	Heading time (days)	±SEM	Tukey
*H. v. spontaneum*	*H. v. spontaneum*	97.9	4.1	a
*H. v. spontaneum*	Heterozygous	103.5	3.7	ab
*H. v. spontaneum*	Hayakiso 2	115.3	4.3	b
Avg. *H. v. spontaneum vrn*-*H1*		105.6		a
Heterozygous	*H. v. spontaneum*	98.1	3.1	a
Heterozygous	Heterozygous	106.9	2.5	ab
Heterozygous	Hayakiso 2	109.5	3.1	b
Avg. heterozygous *vrn*-*H1*	104.8			a
Hayakiso 2	*H. v. spontaneum*	124.7	3.7	a
Hayakiso 2	Heterozygous	140.9	3.8	b
Hayakiso 2	Hayakiso 2	137.9	3.4	b
Avg. Hayakiso 2 *vrn*-*H1*		134.5		b

Different letters in the last column indicate significant differences between the mean values using the Tukey test (*P* < 0.05)

^a^Both *VRN*-*H1* alleles in this population are recessive *vrn*-*H1*

## Discussion

### *HvFT1* increases in copy number are associated with a spring growth habit

Previous studies demonstrated that the BGS213 *HvFT1* allele is sufficient to confer spring growth habit and therefore that it is epistatic to the *VRN*-*H1* and *VRN*-*H2* alleles for winter growth habit (Takahashi and Yasuda 1971; Yan et al. 2006). In the two F_2_ populations reported by Yan et al. (2006) (BGS213 × *H. vulgare* ssp*. spontaneum* and BGS213 × Igri), plants heterozygous or homozygous for the BGS213 *HvFT1* allele had a spring growth habit (average heading time 40 and 59 days, respectively) and the plants homozygous for the *H. vulgare* ssp*. spontaneum* or Igri alleles showed very late flowering or failed to flower (average heading time 106 and >130 days, respectively; Yan et al. 2006). In addition, the introgression of the *HvFT1* early flowering allele from Tammi into the winter-photoperiod sensitive varieties Hayakiso 2 and Dairokkaku 1 also resulted in spring growth habit (Yan et al. 2006). Results from the BGS213 × IMC population in this study confirmed the previous results. Taken together, these experiments indicated that the epistatic effect of the BGS213 *HvFT1* allele on the *VRN*-*H1* and *VRN*-*H2* alleles for winter growth habit is effective across multiple genotypes.

Although the sequences of the coding and flanking regions of the *HvFT1* alleles from barley varieties Morex, IMC, and BGS213 were identical, only the BGS213 allele was sufficient to induce early flowering in the presence of *vrn*-*H1* and *Vrn*-*H2* alleles for winter growth habit. These differences motivated the study of *HvFT1* copy number in these varieties and led to the discovery of the increased *HvFT1* copy number in BGS213. Surprisingly, the BGS213 allele included 4–5 copies of *HvFT1*, which was confirmed in the two backcross introgression lines Hayakiso 2-Tammi and Dairokkaku 1-Tammi (Fig. 1). No *HvFT1* duplications were observed among the other eight varieties characterized for *HvFT1* copy number in this study. This observation, together with the lack of sequence differences among the 4–5 *HvFT1* copies present in the BGS213 allele, suggest that this duplication is of recent origin.

The *HvFT1* allele for spring growth habit found in BGS213 comes from the spring variety Tammi (Olli/Asplund, both spring), which was released in Finland prior to 1950 (received by the National Small Grain Collection in 1949 as PI 175505). This variety can be found in the pedigrees of barley varieties from Finland, Sweden, Canada, and Alaska, where the short growing seasons require varieties with very early flowering and short life cycles.

Additional *HvFT1* alleles dominant for spring growth habit have been reported in barley accessions from North Pakistan, North India, Tibet, and Ethiopia in the germplasm collection at Okayama University (Takahashi 1983). Here, we confirmed the presence of dominant spring *HvFT1* alleles in one accession from Ethiopia (E878) and another one from Russia (U672), and showed that these two alleles were not associated with differences in *HvFT1* copy number (both alleles showed the P_E_ I_TC_ haplotype combination).

With the identification in this study of CNV at the *HvFT1* locus, all four major flowering genes in the *Triticeae* have been reported to have some level of CNV. Two to four copies of the *Photoperiod*-*B1* gene were identified on wheat chromosome 2B and two to three copies of the vernalization gene *VRN*-*A1* were detected recently on wheat chromosome 5A (Díaz et al. 2012). We reported before the presence of CNV of the *ZCCT* genes in both wheat and barley (Dubcovsky et al. 2005; Distelfeld et al. 2009). Other important agronomic genes in the *Triticeae* also show CNV, including a tandem segmental duplication of the wheat dwarfing gene *Rht*-*D1* (Li et al. 2012), and duplications of the frost tolerance *CBF* transcription factors in barley and wheat (Knox et al. 2010). The rapid increase in CNV examples reported in barley and wheat genes likely reflects the relatively high frequency of gene copy number differences in these species. It has been proposed that the abundance of repetitive elements in the large genomes of the *Triticeae* species might be associated with the faster rates of duplications and deletions observed in these species (Dubcovsky and Dvorak 2007; Saintenac et al. 2011; Wicker et al. 2011).

### Effect of increased copy number on gene expression and phenotype

In *Arabidopsis*, which has a small genome and limited numbers of repetitive sequences, copy number variation affects approximately 9 % of the genes (Gan et al. 2011). Many of these CNV are likely pseudogenes since only a small proportion is expressed. Among the 388 expressed genes with CNV, only 54 showed differences in expression that were attributed to CNV, which suggested that CNV has a modest effect on differences in gene expression in *Arabidopsis*. However, several examples of differential expression of genes associated with CNV and phenotypic changes have started to emerge in barley and wheat, suggesting the possibility that the faster rates of duplications (Dubcovsky and Dvorak 2007), together with the strong selection pressures imposed by agriculture may contribute to a larger role of CNV on expression and phenotypic differences in these species than in *Arabidopsis*.

In wheat, the increased copy number observed in the *Ppd*-*B1* locus was associated with significantly higher transcript levels particularly at dawn when expression in the wild type was very low (Díaz et al. 2012). In contrast, the increased copy number of recessive *vrn*-*A1* alleles was associated with slower induction of expression, which is consistent with the increased vernalization requirement and the delayed flowering time observed in the varieties with higher *vrn*-*A1* copy number (Díaz et al. 2012). Large dosage effects of the *VRN*-*H2* locus were observed here in the IMC x Hayakiso 2 segregating population, suggesting that the natural variation in *VRN*-*H2* copy number should also have an effect on flowering time. Natural variation in *VRN2* copy number has been previously described in wheat (Distelfeld et al. 2009). Additional CNV affecting gene expression and phenotype have been reported in genes affecting other important agronomic traits in barley and wheat (Li et al. 2012; Knox et al. 2010; Stockinger et al. 2007; Ragupathy et al. 2008). These examples show that CNV can contribute to important adaptive variation in the *Triticeae* species through changes in the patterns of gene expression.

Using the Hayakiso 2 × Hayakiso 2-Tammi population, we showed that CNV can influence the timing of expression. Plants homozygous for the Tammi *HvFT1* allele (4–5 *HvFT1* copies) had earlier *HvFT1* expression during development than the near isogenic lines with a single *HvFT1* gene. Heterozygous plants (average ~3 copies per genome) showed an intermediate initiation time of *HvFT1* expression levels (Fig. 4) providing additional evidence of the association between *HvFT1* copy number, expression levels and flowering time.

A correlation between earlier increases in *HvFT1* transcript levels and earlier heading time was also observed in wheat. Recombinant substitution lines carrying the *TaFT*-*B1* allele from the variety Hope, which has a retrotransposon insertion in the promoter showed higher *FT1* transcript levels and earlier flowering time than the isogenic lines carrying the wild type *TaFT*-*B1* allele (Yan et al. 2006). Similarly, a winter wheat line transformed with the *TaFT*-*B1* Hope allele showed early flowering even in the absence of vernalization (Yan et al. 2006). To test if the early flowering was associated with CNV we estimated *TaFT*-*B1* copy number in the transgenic lines relative to the non-transgenic Hope allele. Analysis of genomic DNAs from multiple plants from two independent transgenic events (Yan et al. 2006) showed that these transgenic lines have approximately four (3.7 ± 0.27, 8 plants) and five (4.70 ± 0.14, 12 plants) *TaFT*-*B1* copies. Although these differences in copy number may explain the earlier induction of *TaFT*-*B1* in the transgenic wheat plants, we cannot rule out alternative explanations. Since these transgenic events were produced by bombardment, some of the inserted copies may not be functional. Altered transcript levels can be also generated by the truncation of critical regulatory regions in the construct and/or rearrangements during the transformation.

### Haplotype differences in *HvFT1* promoter and first intron

Based on the early flowering associated with the Tammi and BGS213 *HvFT1* alleles, Yan et al. (2006) hypothesized that the I_AG_ intron one haplotype present in these varieties might be associated with their early flowering time, but cautioned that the number of varieties analyzed was insufficient to make valid conclusions. The results from the present study indicate that the early flowering associated with the *HvFT1* alleles from Tammi and BGS213 described by Yan et al. (2006) is most likely the result of their increased copy number. When lines with a single *HvFT1* copy are compared, the intron one I_AG_ haplotype seems to be associated with a delay in flowering relative to the I_TC_ haplotype. In the IMC x Hayakiso 2 population described here, plants homozygous for the I_AG_ haplotype flowered 10–13 days later than the plants heterozygous or homozygous for the I_TC_ haplotype (both P_E_ promoter). Although these differences were not significant (likely due to high variability of the late flowering plants), a similar result was described recently in the cross between the French variety Esterel and the Spanish landrace SBCC016. These two varieties have identical *HvFT1* promoter haplotypes (P_L_) but different intron one haplotypes. In this population, plants homozygous for the I_AG_ haplotype flowered 7 days later (*P* < 0.01) than the plants homozygous for the I_TC_ haplotype (Casas et al. 2011). This result was further validated in a collection of 140 winter barley landraces, in which the I_AG_ haplotype was associated with 6–8 days later flowering than the I_TC_ haplotype and showed a high correlation with latitude (*R* = 0.55; Casas et al. 2011). Cuesta-Marcos et al. (2010) also reported the presence of the I_AG_ haplotype in a winter variety confirming that this haplotype is not associated with early flowering. The association between haplotype differences in the *HvFT1* intron and flowering time is not surprising given the important role of *FT* intron 1 polymorphisms on flowering reported in other species (Adrian et al. 2010; Helliwell et al. 2006; Schwartz et al. 2009; Tiwari et al. 2010). However, a conclusive determination of a causal relationship between the I_TC_ haplotype and early flowering will require experimental validation using transgenic approaches.

Results from this study also showed significant associations between *HvFT1* promoter haplotypes and heading time in lines with a single copy of *HvFT1*, but these differences were not consistent among three populations that used the same *H. vulgare* ssp*. spontaneum* accession as one of the parental lines. Plants carrying the P_E_ haplotype flowered earlier than those carrying the P_L_ haplotype both in the E878 × *H. vulgare* ssp*. spontaneum* and U672 × *H. vulgare* ssp*. spontaneum* segregating populations. However, in the Hayakiso 2 × *H. vulgare* ssp*. spontaneum* population, plants homozygous for the P_E_ haplotype flowered 10–14 days later than those carrying the P_L_ haplotype (*P* < 0.0001). This unexpected result was confirmed by independent experiments in two laboratories. The same trend was observed when the differences between the *HvFT1* alleles were analyzed only within the class homozygous for the *VRN*-*H1* allele from *H. vulgare* ssp*. spontaneum* (Table 4), which was the same accession used in the E878 and U672 populations. This last result indicates that the inconsistent effect of the *HvFT1* P_E_ allele from Hayakiso 2 is not caused by epistatic effects of the *VRN*-*H1* allele.

Taken together, the previous results suggest that the observed differences in the promoter haplotypes are not the cause of the differences in flowering time, but are just markers linked to a yet unknown cause of these differences. The heterogeneous effects of the promoter haplotypes may also explain the limited effect of the promoter haplotypes observed in the study of 140 Spanish winter barley landraces (SBCC; Casas et al. 2011). In this collection, the P_E_ haplotype was associated with 2–3 days earlier flowering in the fall sowing experiment, but with an opposite effect in the April sowing experiment (3.2 days later flowering within the I_AG_ lines). In a subsequent doubled haploid population from the cross SBCC145 (P_E_ I_TC_) x Beatrix (P_L_ I_TC_), the P_E_ haplotype was associated with an earlier heading time, though the differences were significant only within the photoperiod insensitive class (Ponce-Molina et al. 2012). In our E878 (P_E_ I_TC_) × *H. vulgare* ssp*. spontaneum* (P_L_ I_TC_) segregating population, the effects of *HvFT1* on heading time was significant both in photoperiod sensitive and insensitive backgrounds.

### Epistatic interactions between *VRN*-*H1* and *HvFT1* alleles

The heterogeneity of the effects of the promoter haplotypes on heading time was also evident in their epistatic effects. In the E878 × *H. vulgare* ssp*. spontaneum* and U672 × *H. vulgare* ssp*. spontaneum* populations, the plants carrying the *HvFT1* P_E_ I_TC_ haplotype showed a spring growth habit irrespectively of the *VRN*-*H1* allele. This result indicates that this *HvFT1* allele is epistatic to the *VRN*-*H1* and *VRN*-*H2* alleles for winter growth habit present in these populations (Fig. 5). In spite of having an identical *HvFT1* haplotype (P_E_ I_TC_), the Hayakiso 2 allele was not able to overcome the vernalization requirement in the Hayakiso 2 × *H. vulgare* ssp*. spontaneum* population (Table 4). Based on this result, we conclude that the currently known differences in the sequences of the *HvFT1* promoter haplotypes are not sufficient to explain the different epistatic interactions involving this locus.

The increased *HvFT1* copy number found in the BGS213 and Tammi alleles was associated with stronger and more consistent epistatic interactions than the haplotype differences among single copy alleles described above. In the BGS213 × *H. vulgare* ssp*. spontaneum* and BGS213 × Igri populations, the early flowering *HvFT1* allele from BGS213 was epistatic to the alleles for winter growth habit (Yan et al. 2006). Similar epistatic interactions and early flowering were observed when the *HvFT1* allele with increased copy number was introgressed into the varieties Hayakiso 2 and Dairokkaku 1, which have a strong vernalization requirement (Yan et al. 2006), and in the early experiments using the barley variety Tammi (Takahashi and Yasuda 1971). These results indicate that epistatic effects of the *HvFT1* allele from BGS213/Tammi are consistent across different genotypes.

### Conclusions and practical applications

From a practical point of view, our results have clarified the effects of *HvFT1* natural variation on barley flowering time. We demonstrated that the BGS213 genetic stock used to define the dominant *Vrn*-*H3* spring allele includes multiple copies of *HvFT1* that result in earlier expression and earlier flowering time. The large effect of this allele on flowering time may restrict its use to areas or cropping systems that require short growing cycles. In contrast, the allelic differences in *HvFT1* first intron are associated with smaller effects on flowering time that can be used to fine tune flowering time of barley varieties to different or changing environments. The presence of multiple *HvFT1* alleles with diverse effects on flowering time suggests that natural variation at this locus may have contributed to the wide adaptation of barley to different environments.

From a more basic point of view, our results add to a rapidly growing literature on CNV in the *Triticeae* species. CNV has been found for most of the flowering genes studied so far in the temperate grasses, suggesting that this mechanism plays an important role in the generation of novel diversity. This is not surprising given the dynamic nature of the large genomes of the *Triticeae* species that exhibit rates of duplications and deletions several orders of magnitude faster than the rates of nucleotide substitution (Dubcovsky and Dvorak 2007; Saintenac et al. 2011; Wicker et al. 2011). In the long term, gene duplications play an important evolutionary role, as they provide opportunities for diversification and sub-functionalization that can increase adaptative plasticity.
